# Adaptor protein complexes and intracellular transport

**DOI:** 10.1042/BSR20140069

**Published:** 2014-07-29

**Authors:** Sang Yoon Park, Xiaoli Guo

**Affiliations:** *Cell Biology and Metabolism Program, *Eunice Kennedy Shriver* National Institute of Child Health and Human Development, National Institutes of Health, Bethesda, Maryland, 20892, U.S.A.

**Keywords:** adaptor protein complex, ARF1, membrane trafficking, polarized sorting, signal recognition, AMPA, α-amino-3-hydroxy-5-methylisoxazole-4-propionic acid, AP, adaptor protein, APP, amyloid precursor protein, ARF, ADP-ribosylation factors, BFA, brefeldin A, CaSR, calcium-sensing receptor, COPI, coatamer protein I, EGFR, epidermal growth factor receptor, FHH3, familial hypocalciuric hypercalcaemia type 3, HPS, Hermansky–Pudlak syndrome, HSP, hereditary spastic paraplegia, LRO, lysosome-related organelle, MEDNIK, mental retardation, enteropathy, deafness, peripheral neuropathy, ichthyosis and keratodermia, PI4P, phosphatidylinositol 4 phosphate, PIP2, phosphatidylinositol (4,5)-bisphosphate, RE, recycling endosome, SPG, spastic paraplegia, TGN, *trans*-Golgi network, Vps41, vacuolar protein sorting 41

## Abstract

The AP (adaptor protein) complexes are heterotetrameric protein complexes that mediate intracellular membrane trafficking along endocytic and secretory transport pathways. There are five different AP complexes: AP-1, AP-2 and AP-3 are clathrin-associated complexes; whereas AP-4 and AP-5 are not. These five AP complexes localize to different intracellular compartments and mediate membrane trafficking in distinct pathways. They recognize and concentrate cargo proteins into vesicular carriers that mediate transport from a donor membrane to a target organellar membrane. AP complexes play important roles in maintaining the normal physiological function of eukaryotic cells. Dysfunction of AP complexes has been implicated in a variety of inherited disorders, including: MEDNIK (mental retardation, enteropathy, deafness, peripheral neuropathy, ichthyosis and keratodermia) syndrome, Fried syndrome, HPS (Hermansky–Pudlak syndrome) and HSP (hereditary spastic paraplegia).

## INTRODUCTION

**Figure 1:**
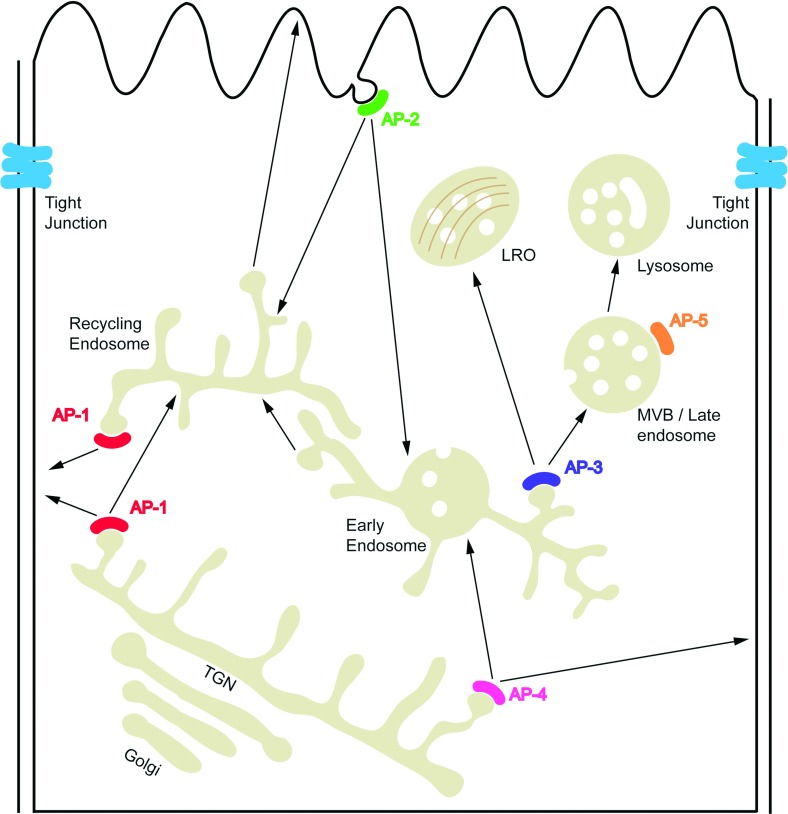
Localization and trafficking of AP complexes AP-1 is localized to TGN and REs, and mediates bidirectional transport between them. AP-1 also mediates basolateral sorting in epithelial cells. AP-2 plays a role in clathrin-dependent endocytosis from the plasma membrane. AP-3 is localized to endosomes, and responsible for the LRO biogenesis. AP-4 is localized to TGN, and mediates vesicle trafficking from TGN to endosomes or basolateral plasma membrane. AP-5 is localized to late endosomes, and its function is largely unknown.

**Figure 2:**
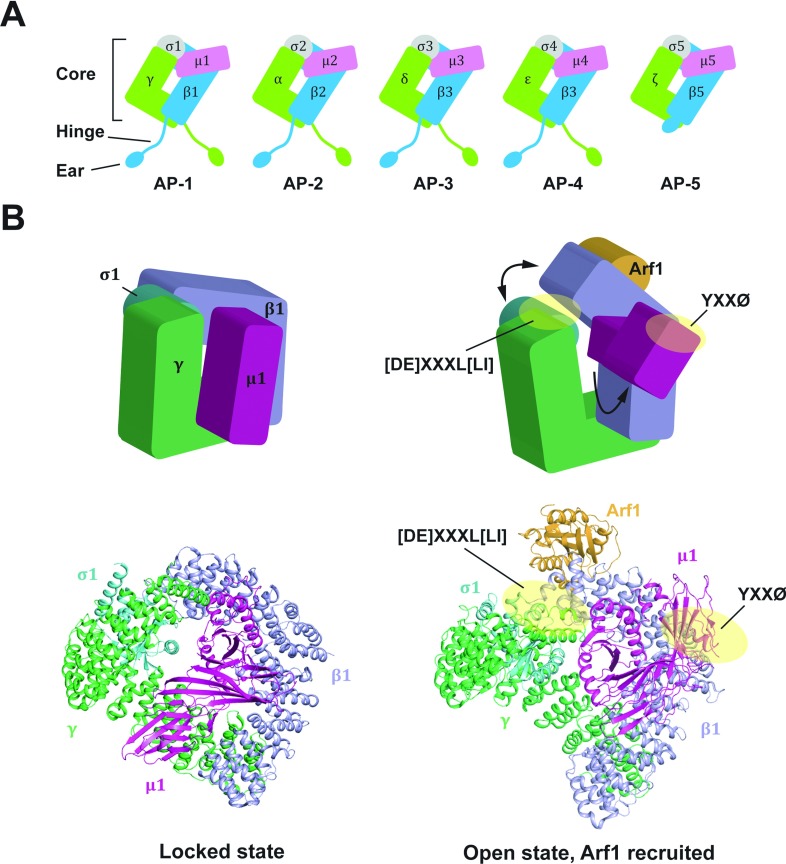
Structures of AP complexes (**A**) Diagrams of heterotetrameric AP complexes. All AP complexes consist of core, hinge, and ear domains, except AP5 that lacks hinge domain. Core domains are responsible for the cargo protein binding and membrane localization. Hinge and ear domains are important to the interaction with coat proteins and regulatory/accessory proteins. (**B**) Locked and open structure of AP-1 core complexes. In the locked state, the cargo-binding sites of AP-1 are hindered by two large subunits (γ and β1). In the presence of Arf1 binding, AP-1 undergoes a large conformational change to the open state. In the open state, both [D/E]XXXL[L/I] and YXXØ-binding sites are exposed and ready to bind to corresponding cargo proteins (yellowish circles). Structures are generated from PDB code 1W63 (locked state) [53], and 4HMY (open state) [15].

In the endocytic and secretory pathways, cargo proteins destined for transport to distinct locations are collectively assembled into vesicles and delivered to their target sites by vesicular trafficking. The AP (adaptor protein) complexes play a critical role in this process. They bind to sorting signals in the cytoplasmic tails of cargo proteins, recruit clathrin and other accessory proteins, and then concentrate the cargo proteins into vesicular carriers, which transport from the donor membrane to the target organelle membrane (Figure 1). Five AP complexes have been identified to date: AP-1, AP-2, AP-3, AP-4 and AP-5, each composed by two large subunits (one each of γ/α/δ/ε/ζ and β1-5, respectively), one medium-sized subunit (μ1–5) and one small-sized subunit (σ1–5) (Figure 2A). Some of these subunits occur as multiple isoforms encoded by different genes: AP-1 has two γ (γ1 and γ2), two μ (μ1A and μ1B) and three σ isoforms (σ1A, σ1B and σ1C); AP-2 has two α isoforms (αA and αC); and AP-3 has two β (β3A and β3B), two μ (μ3A and μ3B) and two σ isoforms (σ3A and σ3B). Combinatorial assembly of different subunit isoforms gives rise to diverse AP heterotetramers, which may display tissue-specific expression and function [1]. The precise molecular and physiological characteristics of the different variants have been studied for some of them and are still lacking for the others. The most studied AP complex variants are for AP-1 and AP-3: AP-1 complexes containing μ1A or μ1B have been referred to as AP-1A or AP-1B, notwithstanding the differences in the other subunits. AP-1A is ubiquitously expressed, whereas AP-1B is epithelial-specific. AP-3 also exists as both ubiquitous and tissue-specific isoforms: AP-3A (δ, β3A, μ3A and σ3) is ubiquitous, whereas AP-3B (δ, β3B, μ3B and σ3) is neuron-specific (Table 1).

The past decade has seen rapid progress in our understanding of protein trafficking mediated by AP complexes. In the present review, we will particularly focus on recent studies concerning the localization, signal recognition, structure and function of the AP complexes.

**Table 1:** AP complexes

Adaptors	Subunits	Localization	Sorting signal	Scaffolds	Lipid binding	GTPase binding	Functions
AP-1	γ1/2	TGN/endosomes	YXXØ	Clathrin	PI4P?	Arf-1	TGN↔endosomes
	β1		[DE]XXXL[LI]				Polarized sorting
	μ1A/B						
	σ1A/B/C						
AP-2	αA/C	Plasma membrane	YXXØ	Clathrin	PIP2	Arf-6?	Clathrin-dependent endocytosis
	β2		[DE]XXXL[LI]				
	μ2						
	σ2						
AP-3	δ	Endosomes	YXXØ	Clathrin?	Unknown	Arf-1	LRO biogenesis
	β3A/B		[DE]XXXL[LI]	Vps41?			
	μ3A/B						
	σ3A/B						
AP-4	ε	TGN	YX[FYL][FL]E	Unknown	Unknown	Arf-1	TGN→endosomes
	β4						Polarized sorting
	μ4						
	σ4						
AP-5	ζ	Late endosomes	Unknown	SPG11/SPG15?	PI3P?	Unknown	Unknown
	β5						
	μ5						
	σ5						

## LOCALIZATION, FUNCTION AND REGULATION OF THE AP COMPLEXES

Transmembrane protein trafficking is a highly dynamic and efficient process regulated by a network of proteins. Over the past decade, AP complexes have become known as key regulators of this process. Each AP complex performs the specific sorting function at distinct intracellular organelles (Figure 1).

AP-2 is the most widely studied family number, which localizes to plasma membrane. AP-2 facilitates clathrin-mediated endocytosis of a wide range of proteins, including receptors, adhesion molecules and viral proteins [2–10]. The selective recruitment of AP-2 to plasma membrane occurs by binding of the α and μ2 subunits to PIP2 [phosphatidylinositol (4,5)-bisphosphate] [11,12]. Arf (ADP-ribosylation factor) 6 may also contribute to the membrane recruitment of AP-2 [13,14] (Table 1).

AP-1 is found on the TGN (*trans*-Golgi network) and endosomes and also associated with clathrin-coated vesicles. Over the past decade, several studies presented evidence that AP-1A localizes to the TGN and endosomes through direct binding to Arf1 and PI4P (phosphatidylinositol 4 phosphate) [15–17]. It mediates the biogenesis of secretory granule [18] and bidirectional transport between TGN and endosomes [16,19]. AP-1B was assumed to localize specifically to REs (recycling endosomes), probably by interaction with Arf6 and PI(3,4,5)P3 [phosphatidylinositol 3,4,5-trisphosphate] [20,21], and to mediate basolateral sorting in epithelial cells [22]. However, using a different tagging method and more advanced microscopy techniques, we recently showed that AP-1A and AP-1B display the same localization. They colocalize to similar extents with TGN and RE markers, as well as with basolateral cargoes transiting biosynthetic and endocytic-recycling routes. We further showed that the membrane recruitment of both AP-1A and AP-1B is sensitive to the Arf–GEF inhibitor BFA (brefeldin A). Moreover, the dominant-negative Arf1 mutant Arf1 T31N, but not the Arf6 mutant Arf6 T27N, disrupts their membrane association [23] (Table 1). The colocalization of AP-1A and AP-1B is consistent with the fact that the main determinants of AP-1 localization to the TGN/REs reside within binding sites for Arf family GTPases on the γ and β1 subunits, and for PI4P on the γ subunit. Furthermore, growing evidence supports the notion that AP-1A also plays an important role in polarized sorting. Gravotta et al. [24] demonstrated that in epithelial cells, knockdown of AP-1A also disrupts polarized sorting. Also in neurons, which do not express AP-1B, disruption of AP-1A causes some somatodendritic proteins, including endogenous NR2A and NR2B, to be missorted to the axon [25]. Taken together, these findings suggest that both AP-1A and AP-1B contribute to the regulation of cell polarity by mediating polarized sorting in biosynthetic and endocytic pathways [26].

Similar to the AP-1 complex, AP-3 and AP-4 also localize to TGN and/or endosomal membranes. And the membrane recruitment of AP-3 and AP-4 are both regulated by Arf1 [27–29]. AP-3 is preferentially localized on a tubular endosomal compartment. Immuno-electron microscopy showed that AP-3 and AP-1 localize to distinct endosomal buds [30,31]. AP-3 mediates cargo transport from tubular endosomes to late endosomes and is involved in the biogenesis of LROs (lysosome-related organelles). It has also been shown that the neuron-specific AP-3B is important for the formation and release of exocytic organelles (large dense core vesicles, synaptic vesicles, etc.) [32–38]. AP-4 is preferentially localized to the TGN and mediates cargo transport from the TGN to endosomes in a clathrin-independent manner [39]. Besides its TGN sorting function, AP-4 is also involved in polarized sorting in epithelial cells and neurons [40,41] (Table 1).

Hirst et al. [42] recently identified an AP-5 complex that is involved in endosomal sorting. AP-5 is localized to late endosomes. Similar to AP-4, AP-5 is not associated with clathrin but instead interacts with SPG11 (spastic paraplegia type 11) and SPG15 (spastic paraplegia type 15) [43]. Both proteins have predicted α-solenoid structures similar to clathrin heavy chain and COPI (coatomer protein complex I) subunits, making them good candidates for the AP-5 scaffold proteins. The membrane recruitment mechanism of AP-5 is also unclear. AP-5 is insensitive to BFA, suggesting that its localization is not regulated by ARF1. One possible explanation is that SPG15 facilitates the membrane docking by interacting with PI3P (phosphatidylinositol 3-phosphate) via its FYVE domain (Table 1).

## SORTING SIGNALS RECOGNIZED BY AP COMPLEXES

The recognition of specific sorting signals in transmembrane cargo by AP complexes confers specificity on membrane trafficking. Sorting signals recognized by AP complexes are located in the cytoplasmic domain. These signals consist of short, linear sequences of amino acid residues. The best-characterized sorting signals are tyrosine-based (YXXØ) and dileucine-based ([DE]XXXL[LI]) signals (X is any amino acid and Ø is a bulky hydrophobic amino acid, i.e. leucine, isoleucine, methionine, valine or phenylalanine), which are recognized by AP-1, AP-2 and AP-3. Tyrosine-based (YXXØ) signals interact with AP complexes through the binding with μ1–μ3 subunits. In contrast, dileucine-based signals bind to the combination of the γ–σ1, α–σ2 and δ–σ3 subunits (Table 1).

In addition to these two types of widely utilized sorting signals, there are also non-canonical signals that can be recognized by AP complexes. A recent study showed that the basolateral sorting signal of TfR, GDNS, contributes to interaction with μ1B [24]. A subset of non-canonical signals, which are not efficiently recognized by μ1A were found to be recognized by μ1B. For example, yeast two-hybrid and GST (glutathione transferases) pull-down experiments showed that the non-canonical tyrosine-based signals and clusters of acidic residues of LDLR that mediate basolateral sorting preferentially bind to μ1B. Thus, the existence of μ1B subunit expands the repertoire of AP-1 signal recognition in epithelial cells [23].

The sorting signals recognized by AP-4 have not been clearly defined until the discovery of the interaction with Alzheimer's disease APP (amyloid precursor protein). A specific and robust interaction was reported between the YKFFE sequence and the AP-4 μ4 subunit. Although the YKFFE sequence fits the minimal consensus for YXXØ signals, it is a distinct type of signal, as mutational and binding analyses revealed that only the Lys residue is unimportant [44] (Table 1). AP-4 μ4 subunit also binds to glutamate receptor δ2. Some non-canonical signals, including the di-aromatic residue (FXF), phenylalanine-based motifs (FGSV) and FR motifs, are required for the binding with μ4 [45].

So far, there is no sorting signal binding to AP-5 has been identified. Actually, the conserved residues binding to YXXØ signals are absent in the μ5 subunit, implicating that AP-5 may bind to other sorting signals in a different way [42] (Table 1).

## STRUCTURAL ASPECTS OF CARGO BINDING OF AP COMPLEXES

In AP complexes, the N-terminal trunk domains of two large subunits, and the full-length μ and σ subunits comprise a ‘core’ domain. The core domain is responsible for many functions, including the recognition of both YXXØ and [DE]XXXL[LI] sorting signals, and membrane recruitment of AP complexes. The C-terminal domains of two large subunits comprise two ‘appendage’ (also called ‘ear’) domains, which play roles in the interaction with regulatory/accessory proteins. These appendage domains are linked to the core domain by two long and mostly unstructured ‘hinge’ domains (Figure 2A). The hinge domains of AP-1, AP-2 and AP-3 subunits have the clathrin-binding motif, which is responsible for the binding to the terminal domain of clathrin heavy chain to form the clathrin-coated vesicles.

In the core domain, the μ subunit is important for the recognition of the YXXØ motif. The structure of the YXXØ motif-binding site was first revealed in the structure of the μ2 subunit complex with the signal peptides of EGFR (epidermal growth factor receptor) and TGN protein TGN38 [46]. The μ2 protein has an elongated, banana-shaped structure that is comprised of 17 β-sheets and organized as subdomains A and B. The sorting signals from EGFR and TGN38 bind to the surface of two parallel β-sheet strands (β1 and β16 in subdomain A), and the hydrophobic pockets, which bind to both tyrosine and Ø residue, exist on both sides of the β16 edge. Additionally, the hydroxyl group of the tyrosine residue interacts with Asp^176^ in the μ2 subunit that is conserved in μ1 and μ3 as well, and is critical for the binding of μ1, μ2 and μ3 to the YXXØ motif. This reflects that μ1 and μ3 bind the signal peptide in a similar manner [46]. Indeed, the structure of the μ1 subunit bound to MHC-I cytosolic tail-HIV-1 Nef fusion protein shows that μ1 also uses the equivalent site on μ2 signal-binding site to bind to a YSQA motif in MHC-I molecule [47]. The structure of μ3 subunit of AP-3 complex was also solved with TGN38 sorting signal peptide, and the binding interface is similar to that on μ2-TGN38 [48]. The μ4 subunit of AP-4 complex, however, has a different mechanism to bind to its sorting signal, which is the non-canonical YX[FYL][FL]E motif. The binding interface of μ4 and the APP sorting signal peptide locates on the opposite face from the μ2 signal-binding site (β4, β5 and β6 in subdomain A) [44]. The structure of μ5 remains to be elucidated.

Another sorting signal in cargo proteins is the [DE]XXXL[LI] motif, which binds to the juncture of γ–σ1, α–σ2 or δ–σ3 subunits in AP-1, -2 or -3, respectively [1]. Although it does not have a canonical acidic residue, the RMpSQIKRLLSE (pS is phosphorylated serine) peptide from CD4 cytosolic tail was co-crystallized with AP-2 core domain. The structure of this complex shows that the di-leucine moiety binds in two adjacent hydrophobic pockets on the σ2 subunit. And glutamine at the -4 position from the first leucine residue of the di-leucine moiety that is commonly occupied by an acidic residue binds to basic patches on both α and σ2 (αR21, σ2R15) with some degree of flexibility [49]. Moreover, the structure of the AP-2 α–σ2 hemicomplex bound to HIV-1 Nef, which has a canonical ENTSLL sorting signal, was elucidated. The ENTSLL signal also binds to hydrophobic pockets of σ2 and basic patches of α–σ2 in same manner with AP-2–CD4 [50]. In addition to the dileucine-motif binding interface, AP-2 α–σ2 hemicomplex has an additional binding interface for Nef. This secondary binding interface is formed between the charged and hydrophobic residues of the α–σ2 hemicomplex and the turn-rich region of the internal loop of Nef. The turn-rich region is anchored internally by a hydrogen bond between the di-leucine motif of Nef and the Nef core. This secondary binding interface is only specific for Nef, and has never been reported with other natural AP-2 cargo proteins [29].

In order to access and bind the cargo proteins, a large conformational change of the AP core domain from a ‘locked’ form to an ‘open’ form is required [12]. In the locked form of the AP-2 complex, the α and β2 trunk domains, the N-terminal domain of μ2, and σ2 form a ‘bowl’-like structure, and the C-terminal domain of μ2, which has an elongated shape, is located at the centre of the bowl. In this form, the YXXØ-binding site on the C-terminal domain of μ2 is buried into the bowl, and the [DE]XXXL[LI]-binding site on α–σ2 is blocked by the N-terminal domain of β2. When the AP-2 complex is recruited to the membrane through PIP2 binding, AP-2 undergoes a conformational change to the open form. In the open form, the bowl structure collapses, and the C-terminal domain of μ2 is expelled and no longer located on the bowl. Also, the N-terminal domain of β2 is displaced, resulting in the exposure of the [DE]XXXL[LI]-binding site on α–σ2. This conformational change allows access of both YXXØ and [DE]XXXL[LI] signals to their corresponding binding sites [12,49,51].

In contrast to the AP-2 complex, phosphoinositide binding (i.e. binding to PI4P) alone is not sufficient for the membrane recruitment of the AP-1 complex, but Arf1 binding is required [19,52]. Moreover, Arf1 not only mediates membrane recruitment, but also facilitates the conformational change of AP-1 from the locked to the open form [15,53] (Figure 2B). In the structure of the AP-1 core–Arf1 complex, Arf1 contains two distinct AP-1 binding interfaces, which include the sites on the canonical switch I and II surfaces on Arf1, and a surface on the C-terminus of Arf1 (‘back side’). In the GTP-bound form of Arf1, the switch I and II surface interacts with α-helices on the β1 subunit (α1, α3 and α5), but in the Arf1–GDP form, the switch I and the β1–α5 are not compatible. Based on the structure of γζ–COP in complex with Arf1 (γ-COP and ζ-COP are two subunits of COPI complex, which are homologous to γ and σ1 subunits of AP-1) [54,55], and other biochemical observations, the switch I and II sites were also found to bind to the γ-subunit of AP-1, although this interaction was not visualized in the crystal structure. Indeed, the Arf1-binding sites on both β1 and γ are important to the membrane recruitment and the subcellular localization of AP-1, but only the β1 site is important for the allosteric activation of AP-1 [15].

## PHYSIOLOGICAL ROLES OF THE AP COMPLEXES

AP complexes function as key regulators of intracellular protein transport. The dysfunction of AP complexes, which interferes with the correct localization of transmembrane proteins, affects a wide variety of cellular processes, including signal transduction, organelle dynamics as well as tissue homeostasis. The physiological roles of AP complexes are impressively illustrated by various inherited diseases and knockout mouse models.

Knockout of γ1 and μ1A subunits in mouse is embryonic lethal, indicating the crucial role of AP-1 in embryonic development [56,57]. However, σ1B-deficient mice are viable and display impaired synaptic vesicle recycling in hippocampal synapses, reduced motor coordination and severely impaired long-term spatial memory, suggesting the important function of σ1B in neurons [58]. The μ1B knockout mice are also viable. The epithelial-specific μ1B-subunit deficiency leads to disrupted polarity and hyperplasia of intestinal epithelial cells. It also causes epithelial immune dysfunction and spontaneous chronic colitis [59,60]. In human, mutation in σ1A subunit causes a neurocutaneous syndrome called MEDNIK (mental retardation, enteropathy, deafness, peripheral neuropathy, ichthyosis and keratodermia) syndrome [61]. Mutation in σ1B subunit causes Fried syndrome (mental retardation, mild facial dysmorphism, calcifications of basal ganglia and hydrocephalus) [62] and Pettigrew syndrome (facial dysmorphism, intellectual disability, Dandy–Walker malformation and inconstant choreoathetosis) [63]. Mutation in σ1C discrupts the endosomal translocation of TLR-3 (toll-like receptor 3) and causes a severe autoinflammatory skin disorder called pustular psoriasis [64]. In addition, patients with Crohn's disease (an inflammatory disease of the intestines) display reduced expression of μ1B [60], and intestinal tumour is associated with down-regulation of μ1B [65]. These tissue-specific phenotypes indicate the non-redundant functions of AP-1 subunits: μ1B functions in epithelial cells; σ1B functions in neurons; σ1C functions in skin; and σ1A may function in both neurons and skin.

Knockout of AP-2 in mouse is also embryonic lethal [66] and missense mutations in σ2 cause FHH3 (familial hypocalciuric hypercalcaemia type 3), an extracellular calcium homoeostasis disorder affecting the parathyroids, kidneys and bone. All three σ2 mutations identified until now in FHH3 patients affect Arg15, which reduce CaSR (calcium-sensing receptor) endocytosis and decrease the sensitivity to extracellular calcium, probably through loss of interaction with a C-terminal CaSR dileucine-based motif [67].

Deficiency of the ubiquitously expressed AP-3A in mouse, for example, β3A mutant *pearl* mice, display coat and eye colour dilution, abnormal LROs, but no neurological defects [68]. Molecular alterations of the β3A subunit in human cause HPS-2 (Hermansky–Pudlak syndrome type 2) [69]. HPS is a disorder characterized by LRO defects, which including oculocutaneous albinism (decreased pigmentation) and bleeding problems (platelet abnormality). In contrast, the neuronal specific AP3B mutant mice show only neurological but not colour defects. For example, the μ3B-deficient mice suffer from spontaneous epileptic seizures [36]. And the β3B knockout mice also display complex neurological and behavioural impairments [70]. The lack of both AP-3A and AP-3B in mocha mice, which are homozygous for a null allele of AP-3 δ subunit, causes the phenotypes observed in both AP-3A- and AP-3B-deficient mice (colour defects, lysosomal abnormalities and neurological defects) [71].

AP-4 β4 knockout mice have no overt abnormalities, except for mild motor neuron impairment. However, they mislocalize AMPA (α-amino-3-hydroxy-5-methylisoxazole-4-propionic acid) receptors and TARPs (transmembrane AMPA receptor regulatory proteins) to autophagosomes in the axons of Purkinje cells and hippocampal neurons [40]. Despite the mild phenotype in mice, mutations of the four subunits of AP-4 in human all caused HSP (hereditary spastic paraplegia), a group of clinically and genetically heterogeneous disorders characterized by lower extremity spasticity and weakness, with or without other neurologic abnormalities [72–76]. Independent groups got this conclusion using different methods, including linkage analysis, microarray analysis or next-generation sequencing, strongly suggesting the important function of AP-4 in neurons.

Until now, no AP-5 knockout mouse model has been established. However, its functional importance was highlighted in a recent study of AP-5 mutations in patients. Notably, mutation in ζ subunit also causes HSP disease [77], indicating AP-4 and AP-5 may sort the same cargo proteins or both important for axonal maintenance [78].

## CONCLUSIONS AND PERSPECTIVES

The past decade has yielded a wealth of new information regarding the localization, function and structure of AP complexes, which has boosted our understanding of the molecular mechanisms that regulate transmembrane protein trafficking. However, many questions remain to be answered. First, the coat protein for AP-3 complex is still poorly understood. There is evidence that AP-3 is associated with clathrin; however, AP3 might be able to function in a clathrin-independent manner. Some genetic and biochemical evidence indicate that AP-3 associates with Vps41 (vacuolar protein sorting 41), which a member of the HOPS (homotypic fusion and vacuole protein sorting) complex. Vps41 self-assembles into a lattice, suggesting that it acts as a coat protein for AP-3 [79–82]. Secondly, some of the AP subunits occur as multiple isoforms. Although some of them have been studied, the exact functions of the other isoforms, for example, γ2, αA and αC, remain to be addressed. Thirdly, the interactions of AP complexes with non-canonical signals need to be further explored. In some cases, these interactions are undetectable or very weak in yeast two-hybrid assay. A new method needs to be developed to identify these interactions. In addition, data to date suggest that AP-1A, AP-1B and AP-4 are all involved in the polarized sorting. However, the mechanisms underlying their roles in polarized sorting are yet to be fully elucidated. For instance, how do these AP complexes work together to maintain the polarity in different tissues? Do they display functional redundancy? Do they have different repertoires of signal recognition? What are the accessory proteins for the polarized sorting and do these proteins work tissue-specifically? Finally, we are only beginning to understand the events regulated by AP-4 and AP-5. Characterization of the motifs that are required for AP-4 and AP-5 binding is a priority. A complete understanding of the function of AP-4 and AP-5 also requires a detailed dissection of structure. Furthermore, elucidation of the cargos sorted by AP-4 and AP-5 will have important implications for the pathogenesis of HSP. Emerging new techniques and technologies, e.g. the advanced microscopes with super resolution or high speed, the CRISPRi technique to efficiently knockout target genes [83] and powerful next generation sequencing technology for genetics study [84,85], are expected to resolve many of the outstanding questions currently confronting cell biologists and help fill the gap of knowledge about these fascinating and highly important proteins.
